# Early life factors and variation in adult kidney function in the Swedish LifeGene cohort

**DOI:** 10.1038/s41598-025-88928-y

**Published:** 2025-02-11

**Authors:** Agne Laucyte-Cibulskiene, Sara Hägg, Anders Christensson, Peter M. Nilsson

**Affiliations:** 1 Department of Clinical Sciences Malmö, Lund University, 202 13 Malmö, Sweden; 2 Department of Nephrology, Skane University Hospital, Ruth Lundskogs gata 14, 214 28 Malmö, Sweden; 3 Department of Medical Epidemiology and Biostatistics, Karolinska Institutet, Stockholm, Sweden

**Keywords:** Birth weight, Early life, Estimated glomerular filtration, Head circumference, Cardiology, Medical research, Nephrology, Risk factors

## Abstract

Intrauterine fetal programming determines cardiorenal interaction later in life. We hypothesize that early life factors affect adult glomerular filtration rate and mean arterial pressure (MAP) directly or by interacting with postnatal growth trajectories. The population-based LifeGene study (Sweden) randomly recruited individuals aged 18 to 43 years (*n* = 12 167). They filled in a web-questionnaire and performed health tests (including bioimpedance measurements). Birth weight (BW), gestational age (GA), head circumference (HC), and birth length data were acquired from the Swedish Medical Birth Register. Postnatal growth was determined from BWz-scores and adult fat mass index. Creatinine and cystatin C-based kidney function were calculated (eGFRcr, eGFRcysC). After adjusting for sex, GA, adult age, and eGFRcr, a 1SD increase in BWz-score predicted a 1.15 mmHg *increase* in MAP. Meanwhile, every 1 cm decrease in HC was associated with an expected 0.29 mL/min/1.73m^2^ decrease in eGFRcr. Lower birth weight-to-placenta ratio was inversely related to eGFRcysC (*p* = 0.034). Postnatal down-regulation significantly affected a relatively lower eGFR but within normal range (*p* < 0.001). The postnatal catch-up did not affect kidney function. This study reveals the complex interrelationship between early life factors and adult kidney function that could be directly and indirectly influenced by adult body fat accumulation.

**Supplementary Information:**

The online version contains supplementary material available at 10.1038/s41598-025-88928-y.

## Introduction

The kidney is a structurally complex organ in the human body that ensures blood filtration, excretion of waste products, regulation of blood pressure, fluid, electrolyte, acid-base balance, hormonal production, etc. In the presence of kidney dysfunction, this homeostatic balance is disrupted; however, signs and symptoms of chronic kidney disease (CKD) manifest later in the process. Identifying and preventing harmful factors that trigger chronic kidney damage is central in treating CKD. Thus, perinatal kidney development and postnatal maturation are pivotal components in the progression and onset of CKD^1^.

Kidney development starts during the first trimester of pregnancy^2^. The number of nephrons after birth, in combination with environmental and genetic factors, determines kidney function and health later in life^3^. The perinatal and postnatal exposures (nutrition, prematurity, low birth weight (BW))^4,5^, and early life exposures to adverse maternal factors (smoking, preeclampsia, hypoxia, etc.)^6,7^, might interrupt the natural development of kidneys and result in reduced kidney volume in adolescence^8^. Low kidney volume increases the susceptibility to kidney dysfunction throughout the lifespan^9,10^.

Overall, intrauterine fetal programming and postnatal growth patterns influence the development of chronic diseases^11^. Early life factors also seem to represent an important etiology for cardiovascular and kidney interactions^12^. We know from studies in adults that increased arterial stiffness and blood pressure could lead to deterioration of kidney function^13^. On the other hand, kidney injury and loss of functioning tissue^14^ alongside glomerular hyperfiltration^15^ might, in turn, cause hypertension and arterial stiffening. Thus, early life exposures might explain this complex crosstalk since reduced fetal growth, low birth weight, and postnatal growth trajectories have been attributed to CKD and kidney failure^4,16,17^, arterial stiffness^18^, and cardiometabolic health^19,20^.

Adiposity might be a mediator linking fetal programming with cardiovascular and kidney outcomes. Fetal origin of increased visceral and total fat mass among children and adults has been acknowledged during the last decades^21–23^. The U-shaped association for which both low and high birth weight are attributed to increased risk for adult obesity has been described^22,23^. It can particularly justify findings contradicting Brenner´s hypothesis^24,25^. For example, when combining birth weight and body composition at different stages of life, large-for-gestational-age (LGA) born children showed a higher risk for obesity and hypertension if their postnatal development did not slow down (no catch-down weight trajectory)^26,27^ Hypothetically, in this population, kidney function decline is secondary to obesity and high blood pressure. Previously, we could show that large for gestational female babies with postnatal down-regulation have worse kidney function in adult life compared to small babies who experience gradual postnatal development^17^. However, no other studies could so far confirm that.

**Fig. 1:**
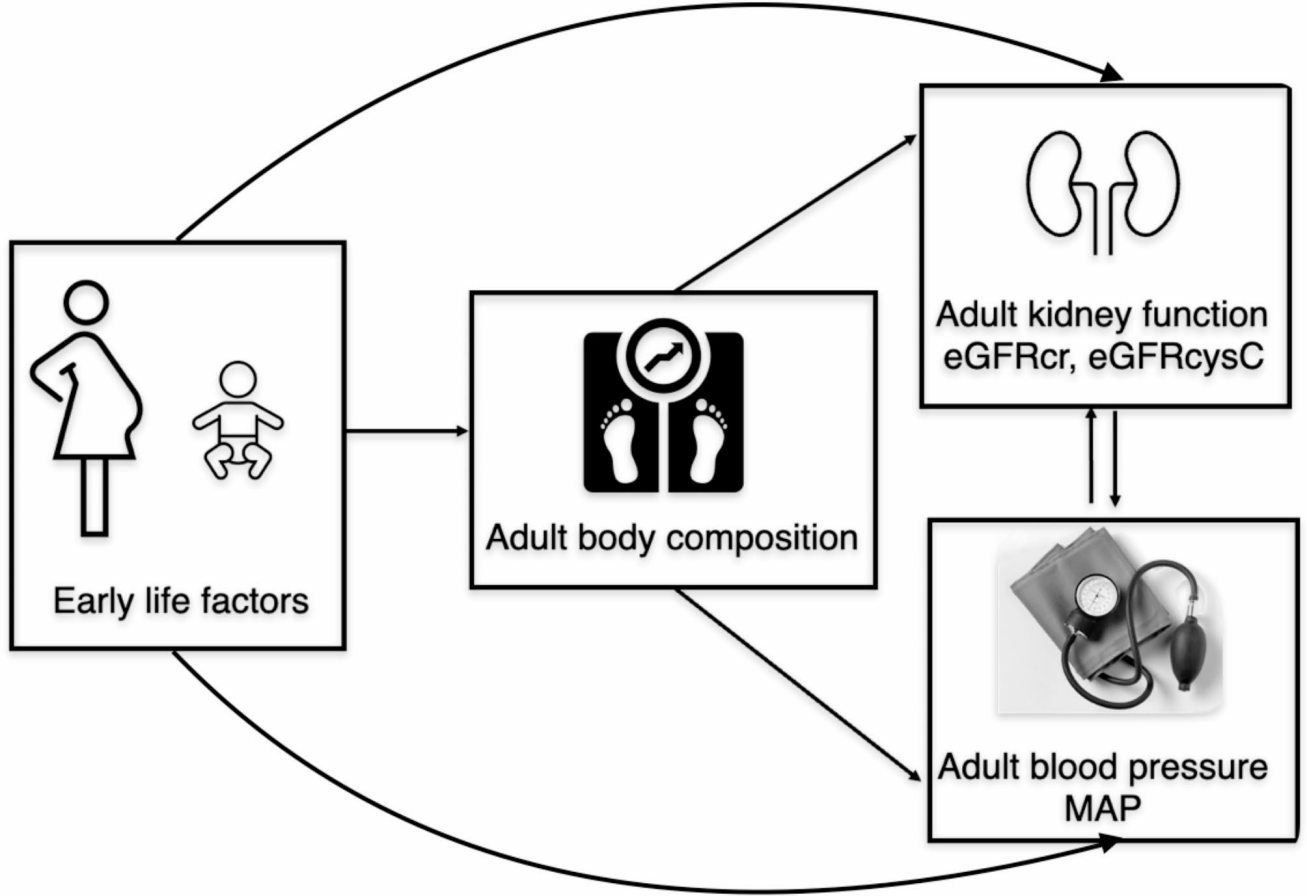
Assumed relationship between early life factors and outcomes. Abbreviations: MAP, mean arterial pressure; eGFR_cr_, estimated creatinine-based glomerular filtration rate; eGFR_cys_, estimated cystatin C-based glomerular filtration rate.

This observational, population-based study focuses on kidney function in younger adults unaffected by advanced ageing and chronic disease burden. Here, we aim to analyze if the same observations apply to individuals aged 18 to 43 years included in the LifeGene study, Sweden. Additionally, we aim to shed light on cardiovascular and kidney interaction by hypothesizing that early life factors either directly affect adult estimated glomerular filtration and mean arterial pressure or via metabolic health (mediators), e.g., body composition (Fig. 1).

## Results

### Descriptive statistics

**Fig. 2:**
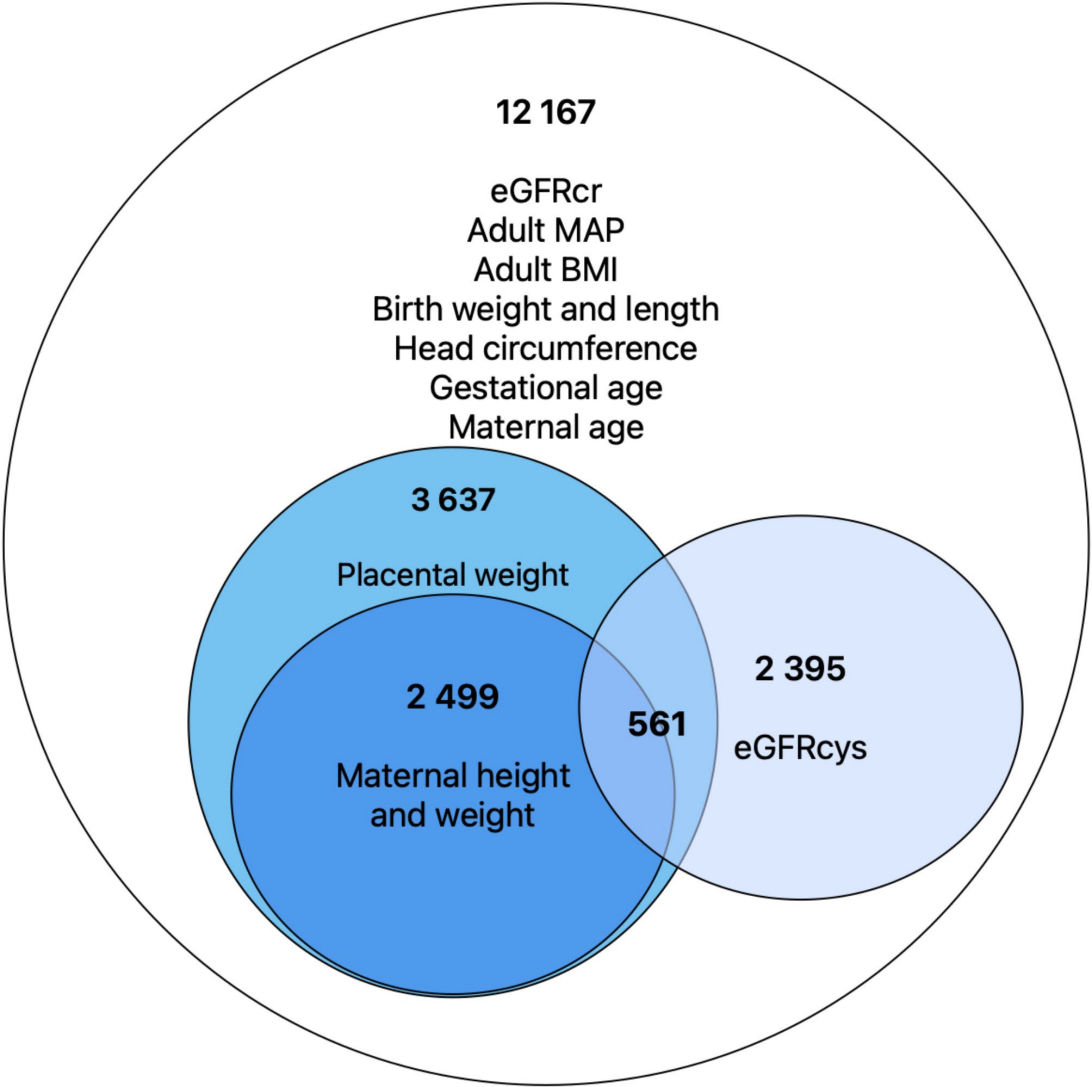
Flow chart of individuals selected. Figure 2 represents the availability of renal function related data in the LifeGene cohort. Abbreviations: BMI, body mass index; MAP, mean arterial pressure; WC, waist circumference; eGFR_cr_, estimated creatinine-based glomerular filtration rate; eGFR_cys_, estimated cystatin-based glomerular filtration rate

A total of 12 167 individuals (42.2% males) who had data on birth-related characteristics (birth weight, head circumference (HC), birth length (BL), gestational age (GA)) and plasma-creatinine levels were analyzed. Of these, 2499 participants also had information on placental weight (PW), maternal height, and maternal body weight before pregnancy and at delivery. However, only 2395 of the total study sample had serum cystatin C measurement. Of these, 561 subjects also had data on placental weight (Fig. 2). Clinical and descriptive characteristics and p-values for differences between men and women are presented in Table 1. Overall, men had significantly higher BW, BL, HC and PW than women (*p* < 0.001). The prevalence of prematurity, SGA and LGA was the same between sexes. Maternal anthropometrics did not differ, nor did the prevalence of diabetes and kidney disease in adult life. Women had significantly higher eGFR (*p* < 0.001 for both eGFR_cr_ and eGFR_cys_) alongside a lower prevalence of CKD (*p* < 0.001). Moreover, women had lower BMI and FMI (*p* < 0.001 for both variables).

**Table 1:** Characteristics of LifeGene study participants. Means, standard deviation (SD), and proportions (percentage).

	Total (*N* = 12 167)	Men (*N* = 5138)	Women (*N* = 7029)	*P*-value
Early life factors				
GA, weeks	40 (2)	40 (2)	40 (2)	0.470
BW, g	3517 (520)	3596 (533)	3458 (503)	< 0.001
BL, cm	50 (2)	51 (2)	50 (2)	< 0.001
HC, cm	35 (2)	35 (2)	34 (2)	< 0.001
BWz	0.02 (1.35)	0.27 (1.35)	-0.17 (1.33)	< 0.001
Prematurity, yes	8.5 (1040)	8.9 (456)	8.3 (584)	0.270
SGA, yes	2.5 (304)	2.4 (122)	2.6 (182)	0.455
LGA, yes	2.5 (304)	2.5 (129)	2.5 (175)	0.939
Placenta weight (PW), g†	614 (141)	630 (150)	603 (134)	< 0.001
BW/PW†	5.9 (1.0)	5.9 (1.0)	5.9 (1.0)	0.616
Maternal age, years	29 (5)	30 (5)	29.4 (4.8)	0.999
Maternal BMI, early pregnancy, kg/m^2^†	21.4 (2.6)	21.5 (2.8)	21.4 (2.6)	0.116
Maternal BMI at delivery, kg/m^2^†	26.4 (3.1)	26.5 (3.2)	26.4 (3.1)	0.137
Maternal weight gain, kg†	13.8 (4.4)	13.9 (4.3)	13.8 (4.3)	0.192
Adult life factors				
Age, years	29 (6)	30 (6)	29 (6)	< 0.001
BMI, kg/m^2^	23.5 (3.3)	24.5 (3.0)	22.8 (3.3)	< 0.001
FMI, kg/m^2^	5.5 (2.5)	4.2 (1.9)	6.5 (2.4)	< 0.001
Smoking^1^				0.165
- Yes	36.8 (4472)	36.0 (3228)	37.3 (4303)	
- No	24.4 (2969)	26.3 (1236)	23.0 (2279)	
- Missing	38.8 (4726)	37.7 (1937)	39.7 (2789)	
Snuff^1^ (nicotine patches)				< 0.001
- Yes	23.7 (2885)	34.7 (1785)	44.4 (110)	
- No	36.3 (4417)	31.3 (1606)	40.0 (2811)	
- Missing	40.0 (4865)	34.0 (1747)	44.4 (3118)	
Diabetes, yes	0.3 (33)	0.4 (18)	0.2 (15)	0.151
Kidney disease, yes	0.7 (91)	0.8 (39)	0.7 (52)	0.903
SBP, mmHg	115 (11)	121 (10)	110 (10)	< 0.001
DBP, mmHg	69 (8)	72 (9)	67 (8)	< 0.001
MAP, mmHg	84 (9)	88 (8)	82 (8)	< 0.001
WC, cm	80 (10)	86 (8)	75 (8)	< 0.001
Kidney function in adult life				
eGFR_cr_, mL/min/1.73m^2^	83 (15)	72 (10)	91 (13)	< 0.001
eGFRcys ‡, mL/min/1.73m^2^	102 (21)	97 (18)	105 (22)	< 0.001
eGFRcr/eGFRcys‡	1.18 (0.27)	1.29 (0.26)	1.12 (0.25)	< 0.001
CKD	4.0 (483)	9.0 (464)	0.3 (19)	< 0.001

Abbreviations: BMI, body mass index; FMI, fat mass index; SBP, systolic blood pressure; DBP, diastolic blood pressure; MAP, mean arterial pressure; WC, waist circumference; GA, gestational age, BW, birth weight; BL, birth length; HC, head circumference; BWz, birth weight sex-specific z-score; SGA, small for gestational age; LGA, large for gestational age; PW, placenta weight; eGFRcr, estimated creatinine-based glomerular filtration rate; eGFRcys, estimated cystatin C based glomerular filtration rate; CKD, chronic kidney disease where eGFRcr is below 60mL/min/1.73m^2^.

Smoking^1^: Question: *Have you ever smoked at least one cigarette?*.

Snuff^1^: Question: *Have you ever used five nicotine patches in your life?*.

†Data in 2 499 individuals, 1482 women and 1017 men.

‡Data in 2 395 individuals, 1461 women and 934 men.

## Association of adult glomerular filtration rate as an outcome with fetal factors

eGFR_cr_ was weakly correlated to BW (*r*=-0.08, *p* < 0.001), BW z-score (-0.09, *p* < 0.001), BL (-0.12, *p* < 0.001), and HC (-0.09, *p* < 0.001), but not GA in the whole cohort. eGFR_cys_ was available in 2 395 individuals but was not correlated to these early life factors.

After performing univariable linear regression analysis with eGFR as an outcome, we found that neither prevalent kidney disease nor diabetes were associated with eGFR levels (*p* > 0.05). Smoking was associated with a decreased GFR_cr_ within the normal range (ß -0.46, 95%CI (-3.52;2.60)) but not eGFR_cys_ (ß 1.68, 95%CI (1.14;2.23)). Snuff was related to lower both eGFR_cr_ (ß -4.45, 95%CI (-5.06;-3.83)) and eGFR_cys_ (ß -4.07, 95%CI (-6.07;-2.07)).

**Fig. 3:**
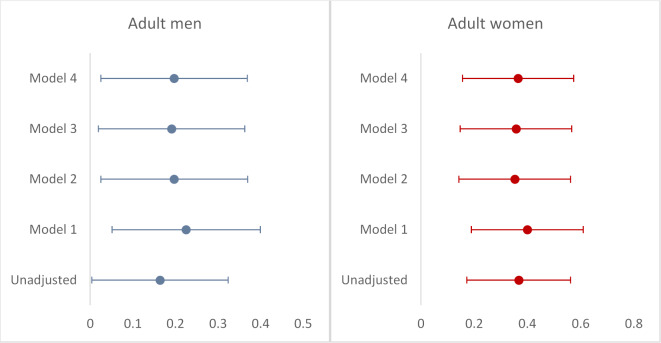
Associations between head circumference and estimated creatinine-based glomerular filtration rate (eGFR_cr_) with and without adjustment for confounders. Forest plots for linear regression models where eGFRcr is a dependent variable. Abbreviations: CI, Confidence Interval. For men: Unadjusted model, *p* = 0.045; Model 1, *p* = 0.012; Model 2, *p* = 0.025; Model 3, *p* = 0,030; Model 4, *p* = 0.024. For women: p-values for all models < 0.001.

Multivariable linear regression revealed that every 1 cm *decrease* in HC was associated with an expected 0.29 mL/min/1.73m^2^*decrease* in eGFR_cr_ in the whole population (*p* < 0.001). In women compared to men, this expected *decrease* in eGFR_cr_ was as follows: 0.37 compared to 0.20 mL/min/1.73m^2^ (*p* < 0.001) (Fig. 3). BW, BW z-score, and BL had no significant relationship with eGFR_cr_ in adjusted *Models 1 to 4* (*p* > 0.05).

**Fig. 4:**
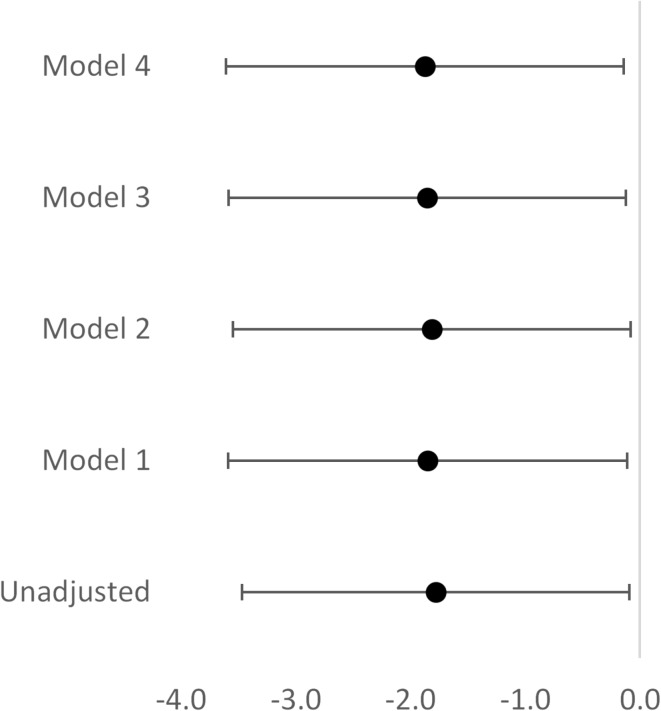
Associations between the birth weight to placenta weight ratio and estimated cystatin C-based glomerular filtration rate (eGFR_cys_) with and without adjustment for confounders in 561 individuals. Forest plot for linear regression models where eGFRcys is a dependent variable. Abbreviations: CI, Confidence Interval. Unadjusted model, *p* = 0.039; Model 1, *p* = 0.037; Model 2, *p* = 0.041; Model 3, *p* = 0.036; Model 4, *p* = 0.034. R^2^ = 1.8% in Model 4.

Placental weight (PW) and eGFR_cr_ were available in 3 637 individuals (1 474 men and 2 163 women). Of these, 561 (195 men and 366 women) had additional data on eGFR_cys_. We observed that PW could neither predict adult eGFR_cr_ nor eGFR_cys_. However, when used as birth weight-to-placenta weight ratio (BW/PW), the higher ratio was associated with a lower eGFR_cys_. It was found that for each 1 unit increase in BW/PW ratio, the expected *decrease* in eGFR_cys_ adjusted for gestational age, sex, adult age, body mass index, mean arterial pressure and smoking/snuff status was 1.87 mL/min/1.73m^2^ (*p* = 0.034) (Fig. 4). These findings were not relevant to eGFR_cr_.

## Association of adult glomerular filtration rate as an outcome with maternal factors

**Fig. 5:**
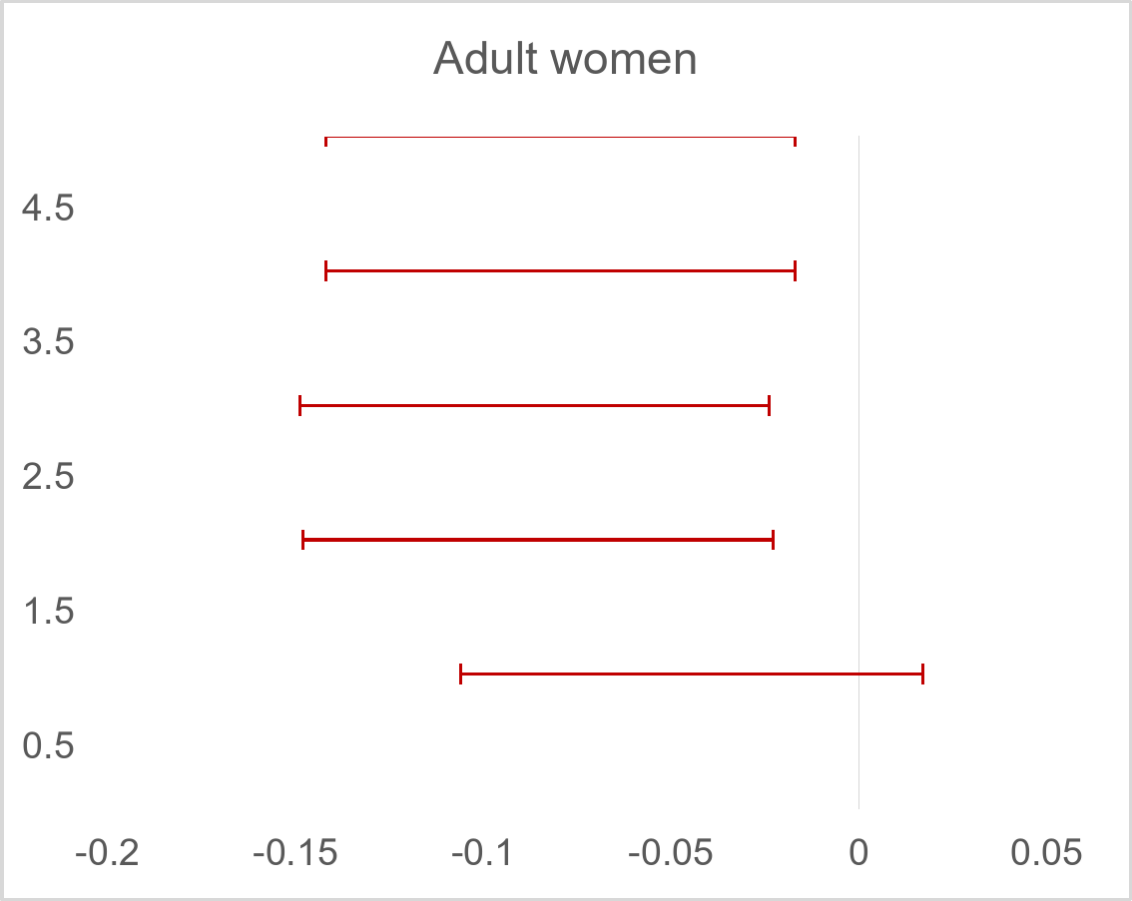
Associations between maternal age during pregnancy and estimated creatinine-based glomerular filtration rate (eGFR_cr_) with and without adjustment for confounders in women. Forest plot for linear regression models where eGFRcr is a dependent variable. Abbreviations: CI, Confidence Interval. Unadjusted model, *p* = 0.159; Model 1, *p* = 0.008; Model 2, *p* = 0.007; Model 3, *p* = 0,013; Model 4, *p* = 0,013. R^2^ = 1.8% in Model 4.

Data on maternal age was available for all study participants. Meanwhile, the maternal body weight at both early pregnancy and delivery was only available in 2 499 individuals and was not associated with adult kidney function in the offspring (unadjusted *p* = 0.625 for eGFR_cys_ and *p* = 0.866 for eGFR_cr_). After adjustments for covariates, a one-year *increase* in the maternal age during pregnancy could predict a 0.08 mL/min/1.73m^2^ *decrease* in eGFR_cr_ in the adult daughters (*p* = 0.013) (Fig. 5). In sons, this association was absent.

## Kidney function and postnatal growth mismatch

In all, 728 individuals (30.5% of them men) were born extremely small (BW z-score more than − 2SD deviation from the mean), and 888 individuals (57.9% of them men) were extremely large (BW z-score deviation than + 2SD away from the mean). Interestingly, small babies had 4.57 mL/min/1.73m^2^*lower* eGFRcr in adult life than the large ones (*p* < 0.001). Cystatin C-based eGFR could not provide this evidence.

A comparison of postnatal growth groups is presented in Table 2. Significantly lower percentage of men was observed in the low BW z-score and lower-than-average adult FMI group (Group 1) (*p* < 0.001). As depicted in Supplemental Figure S1, subjects with high BW z-score but lower-than-average FMI (Group 3) or high BW z-score and higher-than-average FMI (Group 6) had significantly lower eGFR_cr_ as compared to those with low BW z-score and lower-than-average adult FMI (Group 1) (both p values < 0.001), as well as individuals with low BW z-score and higher-than-average adult FMI (Group 4) (*p* < 0.001 and *p* = 0.038, respectively).

**Table 2:** Descriptive characteristics of postnatal mismatch groups.

	Group 1 Low BWz + Lower-than-average FMI (*N* = 392)	Group 2 Normal BWz + Lower-than-average FMI (*N* = 5298)	Group 3 High BWz + Lower-than-average FMI (*N* = 397)	Group 4 Low BWz + Higher-than-average FMI (*N* = 336)	Group 5 Normal BWz + Higher-than-average FMI (*N* = 5257)	Group 6 High BWz + Higher-than-average FMI (*N* = 487)	*P*-value between groups
Early life factors							
GA, weeks	40 (2)	40 (2)	40 (2)	40 (2)	40 (2)	40 (2)	< 0.001
BW, g	2685 (391)	3490 (425)	4420 (353)	2778 (333)	3504 (430)	4388 (351)	< 0.001
BL, cm	48 (2)	50 (2)	53 (2)	48 (2)	50 (2)	53 (2)	< 0.001
HC, cm	33 (2)	35 (2)	36 (2)	33 (2)	35 (2)	36 (1.4)	< 0.001
BWz	-2.66 (0.69)	-0.04(0.96)	2.76 (0.78)	-2.63 (0.62)	-0.01 (0.97)	2.69 (0.70)	< 0.001
Adult life factors							
Age, years	29 (6)	28 (6)	28 (5)	32 (6)	30 (6)	30 (6)	< 0.001
Sex, women	282 (71.9)	3074 (58.0)	161 (40.6)	224 (66.7)	3077 (58.5)	211 (43.3)	< 0.001
BMI, kg/m^2^	20.8 (1.7)	21.4 (1.9)	22.2 (2.0)	25.4 (3.2)	25.5 (3.1)	26.1 (3.2)	< 0.001
FMI, kg/m^2^	4.2 (2.0)	3.9 (2.1)	3.5 (2.2)	6.9 (2.3)	6.8 (2.7)	6.4 (2.9)	< 0.001
Diabetes history, yes	2 (0.5)	11 (0.2)	0 (0.0)	0 (0.0)	17 (0.3)	3 (0.6)	0.280
Kidney disease history, yes	4 (1.0)	27 (0.5)	1 (0.3)	5 (1.5)	48 (0.9)	6 (1.2)	0.039
eGFRcr, mL/min/1.73m^2^	86 (15)	83 (14)	78 (14)	84 (15)	83 (15)	81 (16)	< 0.001
CKD, yes	17 (4.3)	180 (3.4)	20 (5.0)	6 (1.8)	231 (4.4)	29 (6.0)	0.003
SBP, mmHg	111 (11)	113 (11)	114 (11)	114 (11)	116 (11)	117 (11)	< 0.001
DBP, mmHg	67 (8)	68 (8)	68 (9)	69 (9)	70 (9)	71 (9)	< 0.001
MAP, mmHg	82 (8)	83 (8)	83 (8)	84 (9)	85 (9)	86 (8)	< 0.001
WC, cm	72 (6)	75 (7)	78 (7)	83 (10)	85 (10)	88 (10)	< 0.001

Abbreviations: BMI, body mass index; FMI, fat mass index; SBP, systolic blood pressure; DBP, diastolic blood pressure; MAP, mean arterial pressure; WC, waist circumference; GA, gestational age, BW, birth weight; BL, birth length; HC, head circumference; BWz, birth weight sex-specific z-score; eGFRcr, estimated creatinine-based glomerular filtration rate; eGFR_cys_, estimated cystatin C based glomerular filtration rate; CKD, chronic kidney disease where eGFR_cr_ is below 60 mL/min/1.73m^2^.

Except for kidney and body composition parameters, the blood pressure variables were heterogeneous among subgroups Table 2). The individuals with low BW z-score and lower-than-average FMI (Group 1) had significantly lower systolic blood pressure compared to other subgroups and significantly lower diastolic blood pressure in combination with mean arterial pressure as compared to Groups 4 to 6 (all p values < 0.001).

## Mean arterial pressure as an outcome in relation to early life factors and kidney function

In unadjusted linear models, MAP (outcome) was associated with BW, BL, and HC (all p values < 0.001). Interestingly, the BW z-scor2e became significant for predicting MAP after adjusting for sex, gestational age, adult age, and eGFR_cr_ (*p* = 0.022 and 0.021, respectively) (Table 3, Models 2 and 3), but not in further models. Moreover, shorter GA (Table 3, *Models 1 to 5*, all p values < 0.001) and lower eGFR_cr_ (*Models 3 to 5*) were related to higher MAP (all p values < 0.001).

**Table 3:** Factors associated with mean arterial pressure (MAP) as dependent variable. Multivariable linear regression analysis.

Models		ß	SE	95%CI	
				Lower	Upper
Unadjusted	Birth weight z-score	0.415	0.057	0.303	0.526
Model 1	Birth weight z-score	0.035	0.054	-0.070	0.140
	Gestational age, weeks	-0.102	0.042	-0.183	-0.020
	Sex, women	-6.407	0.146	-6.694	-6.120
Model 2	Birth weight z-score	0.122	0.053	0.018	0.226
	Gestational age, weeks	-0.152	0.041	-0.233	-0.072
	Sex, women	-6.075	0.145	-6.359	-5.790
	Age, years	0.241	0.013	0.217	0.266
Model 3	Birth weight z-score	0.122	0.053	0.018	0.225
	Gestational age, weeks	-0.150	0.041	-0.231	-0.070
	Sex, women	-5.360	0.183	-5.719	-5.002
	Age, years	0.233	0.013	0.208	0.258
	eGFRcr, mL/min/1.73m^2^	-0.039	0.006	-0.051	-0.027
Model 4	Birth weight z-score	0.040	0.052	-0.062	0.143
	Gestational age, weeks	-0.153	0.040	-0.232	-0.074
	Sex, women	-4.745	0.183	-5.103	-4.387
	Age, years	0.196	0.013	0.171	0.220
	eGFRcr, mL/min/1.73m^2^	-0.035	0.006	-0.047	-0.023
	Body mass index, kg/m^2^	0.449	0.022	0.405	0.492
Model 5	Birth weight z-score	0.040	0.052	-0.062	0.142
	Gestational age, weeks	-0.153	0.040	-0.232	-0.074
	Sex, women	-4.699	0.184	-5.060	-4.338
	Age, years	0.195	0.013	0.171	0.220
	eGFRcr, mL/min/1.73m^2^	-0.035	0.006	-0.047	-0.023
	Body mass index, kg/m^2^	0.448	0.022	0.405	0.491
	Smoking/snuff, yes	0.439	0.232	-0.017	0.894

Linear regression models with mean arterial pressure as a dependent variable.

Abbreviations: CI, Confidence interval; ß, beta coefficient; SE, standard error; eGFRcr, estimated creatinine-based glomerular filtration rate.

R^2^ = 16.8% in Model 3.

Postnatal mismatch subgroup analysis revealed that in postnatal down-regulation, i.e., high BW z-score and lower-than-average FMI (Group 3), a 1 SD *increase* in BW z-score predicted a 1.15 mmHg *increase* in MAP after adjusting for sex, GA, adult age, and eGFR_cr_ (*p* = 0.025).

As for the relationship between DBP and BW z-score, for each 1 SD *increase* in BW z-score the expected *increase* in DBP is 1.72 mmHg (*p* = 0.002), adjusted for sex, GA, adult age, and eGFR_cr_. SBP was not affected by BW z-score (*p* = 0.758).

The difference in MAP between premature babies and term babies was significant but minor: 84 (±9) vs. 85 (±9) mmHg (*p* = 0.019), respectively.

## Discussion

This population-based study summarizes data on early life factors and its cardiovascular–kidney interactions in over 12,0000 young adults born in Sweden between 1973 and 1998. A smaller head circumference could predict lower creatinine-based kidney function in adult life. In women, the higher birth weight-to-placenta weight ratio was associated with lower cystatin C-based kidney function. In men, no such relationship was observed. We found that a postnatal down-regulation weight trajectory was detrimental to adult kidney function. Meanwhile, the postnatal catch-up pattern was associated with normal kidney function traits. Regarding the bidirectional cardiovascular-renal relationship, the findings indicated that a lower birth weight score was significantly associated with lower MAP – after adjusting for sex, adult age, and creatinine-based kidney function. However, the association was clinically irrelevant.

Cardiovascular-renal interactions are viewed as the cornerstone in the cardiometabolic health and pathogenesis of the so-called cardiorenal syndrome. A plausible mutual origin of disrupted kidney function and cardiovascular development is early life exposures^28^, as previously depicted in Fig. 1. For example, kidneys might specifically be damaged, either directly due to nephron and podocyte endowment^3^ or secondary to accelerated vascular ageing and hypertension^29^. Worsened cardiometabolic health^30–33^, a mediator affecting the cardiovascular-renal continuum, is very likely to be a consequence of deviations in BW and GA or postnatal growth trajectories^30,34^. Establishing this complex cause-effect relationship in adults requires a deeper understanding of how different environmental and socioeconomic factors interact from early life towards adulthood.

Undoubtedly, postnatal growth trajectories influence cardiometabolic health^19,35,36^. Amongst babies born small-for-gestational-age or large-for-gestational-age, if exposed to too fast or too slow postnatal weight gain, the prevalence of chronic diseases in adulthood is increased^30,34^. Postnatal catch-up defines individuals with low BW exposed to postnatal overfeeding, while postnatal down-regulation weight development characterizes high BW but sub-optimal postnatal weight gain^35^. Postnatal growth trajectories influence kidney development since postnatal weight gain from 6 to 24 months shapes the kidney volume^37^. Hence, a postnatal down-regulation is associated with smaller kidneys, and in contrast, postnatal catch-up with larger kidney volume. However, it’s unclear if kidney volume governs earlier manifestation of CKD. Our previous work showed that in a small sample of middle-aged Swedish women (*n* = 94), a higher sex-specific BW z-score in combination with lower than median body mass index (BMI) at 20 years of age was associated with lower cystatin C-based eGFR^17^. Here, we reveal that sex-specific BW z-score above 2 SD in combination with lower-than-average FMI is associated with a decrease in creatinine-based eGFR in young adults. In other words, we confirm that a postnatal down-regulation body weight trajectory is a risk marker for reduced adult kidney function, although within the normal range. Regarding the individuals with sex-specific BW z-score below − 2 SD in combination with higher-than-average FMI (postnatal catch-up), the significantly higher creatinine-based kidney function as compared to postnatal down-regulation group (*p* < 0.001) could reflect glomerular hyperfiltration, which is per se a marker of future cardiometabolic risk^15,38,39^. Hence, these individuals should not be misclassified and would benefit from a more precise measurement of the glomerular filtration rate with iohexol plasma clearance^40^.

The significant positive relationship between birth weight and MAP observed in this study is a subject of debate. We show that the higher BW z-score could predict the rise in adult MAP and DBP independently of kidney function. These findings contradict the proposed Brenner´s hypothesis. We know from previous reports^4,41–43^ that adults with a history of low birth weight or born preterm have higher blood pressure. Nevertheless, the opposite association has also been recognized. For example, according to a recent meta-analysis^44^, higher BW determines predisposition to higher DBP in an age-dependent manner. Obesity mediates this cross-talk, especially if large for gestational age born babies develop obesity during childhood^26,27^. Although the findings show that the association between BW z-score and MAP in the postnatal down-regulation is significant, the effect (1.15 mmHg increase in MAP) is very small and thus clinically irrelevant.

The observed smaller HC in association with reduced adult kidney function in subjects studied, supposedly reflects an unfavorable intrauterine environment. Adverse maternal factors mediate chronic intrauterine hypoxia and cardiac overload, thus causing blood redistribution to essential organs^37^. It was demonstrated by Montaldo et al.^45^ that neonates with intrauterine growth restriction (IUGR) compared to normal neonates were exposed to decreased postnatal regional cerebral and renal oxygenation. Moreover, IUGR was related to higher prevalence of microalbuminuria alongside elevated neutrophil gelatinase-associated lipocalin (NGAL) – a marker of acute kidney injury. Kooijman et al.^46^ could associate preferential fetal blood flow redistribution to the brain with kidney volume at 5.9 years of age; however, not linked to kidney dysfunction or albuminuria^46^. A report from the Netherlands^37^ showed that lower third-trimester HC, in concordance with abdominal circumference, was associated with reduced combined and relative kidney volume at two years of age. Since head circumference (HC) is also a proxy of maternal nutrition and lifestyle habits^47–49^ our findings should be analyzed considering Swedish maternal care quality and environmental factors between 1973 and 1998. Firstly, Nordic Nutritional Recommendations were launched in 1980 and updated several times^50^ resulting in diverse dietary recommendations in pregnancy. Previous reports show that maternal undernutrition is associated with worse kidney outcomes^51^ and altered neurodevelopment^52^ secondary to intrauterine micronutrient, energy, and protein deficits. Similarly, maternal obesity is also responsible for altered fetal brain and kidney development^52,53^. Secondly, a decreasing prevalence of maternal smoking was documented from 1983 to 1992^54^. Smoking has been attributed to smaller HC in Swedes^49^ and smaller infant kidneys in the Netherlands^55^. Finally, climate change and variability and increasing air pollution trends due to urbanization might also influence our findings^56–58^.

Placental failure to ensure sufficient nutrition results in a discrepancy between BW and PW^59^. Typically, the BW-to-PW ratio reaches 5 to 7 at delivery^60^. A hypothesis that is currently debated proposes that smaller babies with relatively larger placentas might represent placenta insufficiency and increase the risk for fetal death^59^. However, a Norwegian report^61^ could confirm the opposite, i.e., that much larger babies relative to PW are exposed to a higher relative risk for fetal death. We could previously show that a lower BW-to-PW ratio predicts higher creatinine-based eGFR in middle-aged men^17^. We could also observe that this finding might even be related to lower cystatin C-based eGFR in younger adults. Unfortunately, this variable was only available in a small subset of the LifeGene cohort. These results should be carefully considered since the average eGFR_cys_ was 103 (59 to 189) mL/min/1.73^2^ and the average eGFR_cr_ 89 (57 to 146) mL/min/1.73^2^, i.e., within the normal range.

The study limitations should be highlighted. *Firstly*, this study enrolled mostly Swedish-born subjects from the Stockholm Region; therefore, it is hard to generalize for the global population. The maternal country/region of origin was diverse (Supplemental Table S2), but it mainly consisted of mothers born in Sweden. *Secondly*, the analysis of serum cystatin C was performed between 2009 and 2010, before the worldwide introduction of a calibrated method^62^, and therefore a slight deviation in cystatin C-based eGFR might exist. As expected, eGFRcr and eGFRcys demonstrate different associations to several parameters. These two GFR markers show different results which most often is dependent of body composition, e.g. muscle mass. This fact makes the conclusions less sharp. *Thirdly*, not all birth variables and eGFR_cys_ data were available in the participants studied plausibly affecting predictive effects. *Fourthly*, temporal aspects of body composition could not be addressed properly due to the lack of data representing BMI or FMI variation through the lifetime. *Fifthly*, manual measurement of office blood pressure was utilized. Either 24-hour ambulatory blood pressure measurement or standardized home blood pressure measurement would provide more accurate information on hemodynamic regulation. *Lastly*, the absence of data on albuminuria limits the identification of kidney damage that manifests without a significant decline in eGFR. Other urinary markers, as well as urinary proteomics, could be of value in better defining early life influences on renal function.

The strength of this work is analysis of younger individuals in a sample size of over 12,000 individuals allows the identification of early stages of organ damage. Preventive medicine is very important due to population aging and aims to moderate the chronic disease burden trends worldwide. The high quality of birth data was also ensured by using the Swedish Medical Birth Register for LifeGene subjects born in 1973 or thereafter^63^. Inconsistent findings of studies clarifying the link between early life anthropometry and non-communicable diseases are widely discussed in the meta-analysis by Brander et al.^64^. The use of body composition over BMI and adjustment for current body size in regression analysis are critical in exploring the link between perinatal and postnatal factors and risk of adult chronic diseases^64^.

In conclusion, smaller head circumference is associated with lower estimated creatinine-based GFR in adult life. A lower birth weight-to-placenta ratio is also related to lower estimated cystatin C-based GFR. The postnatal weight down-regulation trajectory significantly affects a relatively lower eGFR but within the normal range. However, the postnatal catch-up pattern is associated with unaffected kidney function in young adults. The relationship between lower birth weight z-score and lower adult mean arterial and diastolic blood pressure was statistically but not clinically significant. This study reveals the complex interrelationship between early life factors and adult kidney function that could be directly and indirectly influenced by body fat accumulation.

## Methods

### Study sample

The LifeGene study randomly invited individuals 18 years or older from the general population in Stockholm, Sweden, between 2009 and 2016^65^. The participants completed a comprehensive web-based questionnaire consisting of nine main parts: sociodemographic variables, lifestyle, women’s health, self-care, living conditions, health history, mental health, asthma or allergies, and injuries. A total of 12 167 individuals from the LifeGene Study between the ages of 18 and 43 were included in this study. Exclusion criteria: absence of data on birth weight, length and head circumference, gestational age, maternal age, body composition, blood pressure, and plasma creatinine.

All participants signed informed consent forms either at the site for blood sample collection or electronically. The ethical principles applied in this study was in line with The World Medical Association Declaration of Helsinki. Non-fasting blood samples were collected.

The study data were linked with the Swedish Medical Birth Register^63^, launched in 1973, which collects data on early life factors. In contrast, the registration of maternal data (weight, body mass index and smoking habits) started in 1983.

### Study variables

Variables and data sources utilized for this study are listed in Supplemental Table S1.

### Postnatal growth mismatch

The postnatal growth pattern was defined from BW z-score and bioimpedance acquired fat mass index (FMI). We have especially focused on two main features – *postnatal down-regulation* and *postnatal catch-up*. *Postnatal down-regulation* represents babies that are born larger in size than expected for gestational age but, during the postnatal period, develop at a slower pace than their counterparts. *Postnatal catch-up* defines a combination of poor fetal growth with rapid postnatal growth dramatically altering cardiometabolic health^36,66^.

FMI was used to explore the postnatal growth curve because it performed better in defining body composition and metabolic health than body mass index (BMI)^67^ in non-obese LifeGene Study participants.

The birth weight (BW) z-scores were calculated according to intrauterine growth curves for boys and girls^68^, as described in more detail in statistical part. *Low BWz* corresponded to more than − 2SD deviation from the mean, *normal BWz* - +/-2SD from the mean, and *high BWz* is a deviation more than + 2SD from the mean. Adult FMI was categorized as *lower-than-average FMI* – below sex-specific average, 6.1 kg/m^2^ for women, and 3.9 kg/m^2^ for men; *higher-than-average FMI* – equal or above sex-specific average.

The study sample was then divided into six groups:

1: Low BWz and lower-than-average FMI;

2: Normal BWz and lower-than-average FMI;

3: High BWz and lower-than-average FMI (*postnatal down-regulation*);

4: Low BWz and higher-than-average FMI (*postnatal catch-up*);

5: Normal BWz and higher-than-average FMI;

6: High BWz and higher-than-average FMI.

### Definition of kidney function

Estimated glomerular filtration rate (eGFR) formulas were employed: i.e., cystatin C eGFR equation based on Caucasian, Asian, pediatric, and adult cohorts (CAPA, eGFR_cys_)^69^ and the Lund-Malmö revised creatinine-based eGFR equation (LMrev, eGFR_cr_)^70^. Both equations are validated and recognized by KDIGO 2024^71^. The latter eGFR equation is widely used in Sweden since it is validated^72^ and is superior to other creatinine-based equations in defining kidney function.

### Statistical analysis

Continuous normally distributed variables are presented as means with standard deviation (SD), skewed data – as median with interquartile range (IQR), and categorical variables – as frequencies with percentages. The birth weight (BW) z-scores were calculated as described below^68^.

The equation for expected BW:

*For boys:*

$$\:Expected\:BW=\:-\text{1,907345}\:x\:10\text{-6}\:x\:GA\text{4}\:+\:\text{1,140644}\:x\:10\text{-3}\:x\:GA\text{3}-\:\text{1,336265}x\:10\text{-1}\:x\:GA\text{2}+\text{1,976961}x\:GA\:+\:\text{2,410053}x\:10\text{2}\:$$

*For girls:*

$$\:Expected\:BW\:=\:-2.761948\:x\:10\text{-6}\:x\:GA\text{4}\:+\:1.74484\:x\:10\text{-3}\text{}x\:GA\text{3}\:\--\:2.893626\:x\:10\text{-1}\:x\:GA\text{2}+18.91197\:x\:GA\:\--\:4.135122\:x\:10\text{2}$$

The equation BW z-score:

$$\:BW\:z-score=\frac{BW\left(measured\right)-BW\:\left(expected\right)}{SD\left(expected\right)}$$

Where BW(measured) is a raw value, BW(expected) is the calculated value, and SD(expected) is the population standard deviation.

For researching cardiovascular and renal interaction we utilized linear regression analysis with two different outcomes: (1) eGFR (creatinine or cystatin C-based ), and (2) mean arterial pressure (MAP). First, the univariate regression analysis with variables listed in Supplemental Table S1 was employed. Then, maternal and fetal factors (as explanatory variables) were adjusted for covariates such as adult age, sex, body mass index, and smoking/snuff status.

The linear regression models for eGFR as an outcome:

- Model 1: eGFR (outcome) ~ *early life factor* adjusted for sex and gestational age.
- Model 2: + adjustment for adult age and body mass index.
- Model 3: + adjustment for mean arterial pressure.
- Model 4: + adjustment for smoking/snuff status.

The linear regression models for MAP as an outcome:

- Model 1: MAP (outcome) ~ birth weight z-score adjusted for sex and gestational age.
- Model 2: + adjustment for adult age.
- Model 3: + adjustment for eGFR.
- Model 4: + adjustment for body mass index.
- Model 5: + adjustment for smoking/snuff status.

To measure the amount of multicollinearity in linear models, the Variation Inflation Factors (VIF) were calculated. Variables with VIF levels above 2.0 were excluded from the models. Statistical analysis was performed with IBM SPSS Statistics software (Version 29.0, Chicago, IL, USA) and Microsoft^®^ Excel for Mac (Version 16.88, Microsoft Corporation, 2021). P values below 0.05 were considered significant.

## Electronic supplementary material

Below is the link to the electronic supplementary material.

Supplementary Material 1

## Data Availability

Due to Swedish laws on personal integrity and health data, as well as the Ethics Review Board, we are not allowed to make any data, including health variables, open to the public, even if made anonymous. The data could be shared with other researchers after a request to the contact persons for The LifeGene study – Prof. Nancy Pedersen, nancy.pedersen@ki.se, or Dr. Sara Hagg, sara.hagg@ki.se. For details about the LifeGene cohort and instructions on how to apply for data, see link: www.lifegene.se.
