# Supplementary material for: Early life factors and variation in adult kidney function in the Swedish LifeGene cohort
Source: Sci Rep. 2025 Feb 11;15:5046. doi: 10.1038/s41598-025-88928-y (PMC11814342; doi:10.1038/s41598-025-88928-y)
Supplement: Supplementary file 1 — Supplementary Material 1 [file 41598_2025_88928_MOESM1_ESM.pdf]

Supplemental materials

Supplemental Figure S1 Mean values of creatinine-based glomerular filtration rate ($eGFR_{cr}$) in postnatal mismatch subgroups.

Supplemental Table S1. Description of study variables and data sources

Variable		Assessment method	Data source
Anthropometric data	height (m), waist circumference (WC, cm)	manual tape	The LifeGene Study dataset [21]
	weight (kg)	calibrated scale	
	body mass index (BMI)	weight(kg)/height(m)^2	
Bioimpedance body composition analysis	fat mass (kg)	bioimpedance analysis, Tanita BC-418 MA, Japan	
	fat mass index (FMI)	fat mass(kg)/height(m)^2; bioimpedance analysis, Tanita BC-418 MA, Japan	
Blood pressure	systolic blood pressure (mmHg), diastolic blood pressure (mmHg)	manual device, performed by trained medical personnel	
Blood samples	plasma creatinine (mmoL/L) (Li-Hep with gel plug), serum cystatin C (mg/L) (Li-Hep with gel plug)	Unilabs, St. Göran Hospital, Stockholm, Sweden, and Department of Clinical Chemistry, Karolinska University Hospital, Stockholm, Sweden. Samples analyzed during the period from 2009-10-07 until 2010-03-31	
Socio-demographic and lifestyle data	age, sex, smoking, and snuff habits (current, former, non-smoker)	web-questionnaire	
Fetal early life factors	birth weight (g) (BW), birth length (cm) (BL), head circumference (cm)	calibrated scale and manual tape, performed by a midwife	The Swedish Medical

	(HC), placenta weight (g) (PW)		Birth Register [22]
	gestational age (weeks) (GA)	estimated either by date of the last menstruation period, by ultrasound when available, or from a clinical record at birth	
Maternal factors	maternal age at delivery	calculated from the first six digits in mother's personal identity number	
	maternal weight (kg) at pregnancy start and delivery	measured with a calibrated scale when wearing light indoor clothes	
	maternal height (cm)	self-reported	
	maternal weight gain during pregnancy	weight gain (kg) = weight at delivery (kg) – weight at pregnancy beginning (kg)	

Supplemental Table S2. Maternal country/region of birth.

Country/region of birth	Frequency	Percent
Swedish	10797	88.7
Other Scandinavian countries	615	5.1
African	45	0.4
Asian	199	1.6
European	341	2.8
North American	36	0.3
South American	69	0.6
Former Soviet Union	21	0.2
Oceania	2	0.0
Other	9	0.1
Missing data	33	0.3
Total	12167	100.0

Supplemental Figure 1. Mean values of creatinine-based glomerular filtration rate ($eGFR_{cr}$) in postnatal mismatch subgroups.

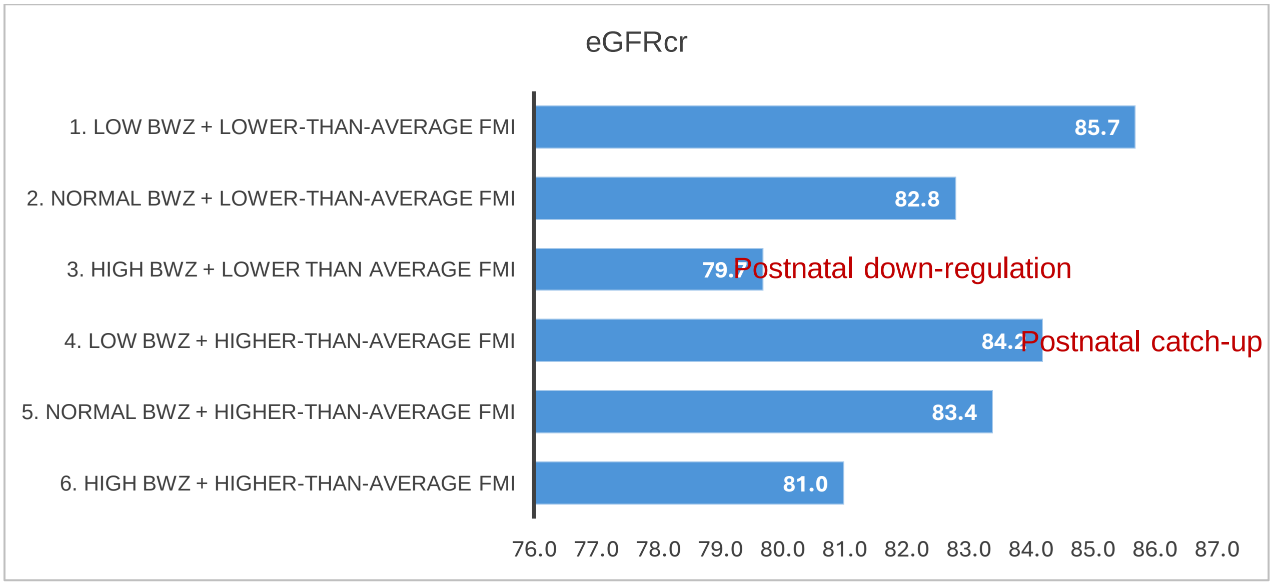

Abbreviations**:** eGFRcr, estimated creatinine-based glomerular filtration rate; BWz, birth weight z-score; FMI, fat mass index. P- values for group comparison: group 1 vs group 2, p=0.001; group 1 vs group 3, p<0.001; group 1 vs group 4, p=0.933; group 1 vs group 5, p=0.047; group 1 vs group 6, p<0.001; group 2 vs group 3, p<0.001; group 2 vs group 4, p=0.804; group 2 vs group 5, p=0.415; group 2 vs group 6, p=0.143; group 3 vs group 4, p<0.001; group 3 vs group 5, p<0.001; group 3 vs group 6, p=0.966; group 4 vs group 5, p=0.999; group 4 vs group 6, p=0.038; group 5 vs group 6, p=0.008.
